# HIV-1, HBV, HCV, HTLV, HPV-16/18, and *Treponema pallidum* Infections in a Sample of Brazilian Men Who Have Sex with Men

**DOI:** 10.1371/journal.pone.0102676

**Published:** 2014-08-01

**Authors:** Caroline C. Soares, Ingebourg Georg, Elisabeth Lampe, Lia Lewis, Mariza G. Morgado, Alcina F. Nicol, Adriana A. Pinho, Regina C. S. Salles, Sylvia L. M. Teixeira, Ana Carolina P. Vicente, Raphael P. Viscidi, Selma A. Gomes

**Affiliations:** 1 Laboratório de Virologia Molecular, IOC, Fiocruz, Rio de Janeiro, Brasil; 2 Laboratório de Análises Clínicas, Seção Imunodiagnóstico, IPEC, Fiocruz, Rio de Janeiro, Brasil; 3 Laboratório de Referência Nacional para Hepatites Virais, IOC, Fiocruz, Rio de Janeiro, Brasil; 4 Laboratório de AIDS e Imunologia Molecular, IOC, Fiocruz, Rio de Janeiro, Brasil; 5 Laboratório Interdisciplinar de Pesquisas Médicas, IOC, Fiocruz, Rio de Janeiro, Brasil; 6 Pós-doutoranda do laboratório de Educação em Ambiente e Saúde do Instituto Oswaldo Cruz, Fiocruz, Rio de Janeiro, Brasil; 7 Laboratório Municipal de Patologia Clínica de Campinas, São Paulo, Brasil; 8 Laboratório de Genética Molecular e Microrganismos, IOC, Fiocruz, Rio de Janeiro, Brasil; 9 Department of Pediatrics, Johns Hopkins University School of Medicine, Baltimore, Maryland, United States of America; Centers for Disease Control and Prevention, United States of America

## Abstract

**Background:**

Men who have sex with men (MSM) are more vulnerable to blood-borne infections and/or sexually-transmitted infections (STI). This study was conducted to estimate the prevalences of mono and co-infections of HIV-1 and other blood-borne/STIs in a sample of MSM in Campinas, Brazil.

**Methods:**

Responding Driven Sampling (RDS) was used for recruitment of MSM. Serum samples collected from 558 MSM were analyzed for the presence of serological markers for HIV-1, HBV, HCV, HTLV, HPV-16/18, and T. pallidum infections.

**Results:**

The highest prevalences of infection in serum samples were found for HPV-16 and 18 (31.9% and 20.3%, respectively). Approximately 8% of the study population showed infection with HIV-1, and within that group, 27.5% had recently become infected with HIV-1. HBV infection and syphilis were detected in 11.4% and 10% of the study population, respectively, and the rates of HTLV and HCV infection were 1.5% and 1%, respectively. With the exception of HTLV, all other studied infections were usually found as co-infections rather then mono-infections. The rates of co-infection for HCV, HPV-18, and HIV-1 were the highest among the studied infections (100%, 83%, and 85%, respectively). Interestingly, HTLV infection was usually found as a mono-infection in the study group, whereas HCV was found only as a co-infection.

**Conclusions:**

The present findings highlight the need to educate the MSM population concerning their risk for STIs infections and methods of prevention. Campaigns to encourage vaccination against HBV and HPV could decrease the rates of these infections in MSM.

## Background

Men who have sex with men (MSM) are at an increased risk of blood-borne infections and/or sexually-transmitted infections (STIs). HIV-1 is one of the most important emergent pathogens in the last century, and in Brazil, ∼630,000 individuals are HIV-1 infected [1], [2]. While the prevalence of HIV-1 infection is <1% in the general population of Brazil, the prevalence in MSM is estimated to be >10-fold greater. Syphilis, an STD (sexually transmitted disease) caused by *Treponema pallidum* (TP), is found in ∼0.5% of the overall Brazilian population [3]. Hepatitis B virus (HBV) and hepatitis C virus (HCV) are the major etiological agents of chronic hepatitis, cirrhosis, and hepatocellular carcinoma, and the World Health Organization has estimated that ∼240 million people worldwide are chronic carriers of HBV. This number is ∼3-fold greater and 10-fold greater than the estimated numbers of chronic HCV and HIV-1 carriers, respectively. In Brazil, the prevalence of HBV infection among blood donors ranges from 3.7% to 11.1%, and <1% of the general population is infected by HCV [4]. HBV transmission occurs mainly by percutaneous or mucosal exposure to infected blood, but also occurs through perinatal exposure and sexual intercourse [5]. HCV is essentially a parenterally (blood contact) transmitted virus. Surprisingly, there is little evidence for sexual transmission of HCV, even though HCV shares routes of transmission used by HBV and HIV-1, which are transmitted mainly by sexual and parenteral exposure [6].

Human T-cell lymphotropic virus (HTLV) was the first oncogenic retrovirus discovered in humans, and four HTLV types (1–4) have been identified. HTLV-1 is the etiological agent for adult T-cell leukemia/lymphoma, tropical spastic paraparesis/HTLV-1-associated myelopathy, and other inflammatory diseases. Thus far, no effective treatment has been developed for these progressive and chronic diseases. HTLV has demonstrated three routes of transmission: (i) from mother to child during prolonged breastfeeding, (ii) by contact between sexual partners, and (iii) through blood transfusion. It is estimated that 10–20 million people are infected with HTLV-1/2 types worldwide, and in Brazil, where these virus infections are endemic, ∼2–3 million people are seropositive. Due to the high prevalence of HTLV infection, blood donor screening became mandatory in Brazil and several other countries during the 1990's [7].

Human papillomavirus (HPV) infections are among the most common sexually transmitted infections worldwide, and are a leading cause of anogenital malignancies. The incidence of anal cancer is particularly high among HIV-1-positive individuals, MSM, and transplant recipients [8]. HPV-16 is responsible for ∼60% of HPV-associated penile cancers, and seropositivity to HPVs 18 and 51 is associated with anal cancer [9], [10], [11]. Not all individuals infected with HPV by natural exposure develop a detectable antibody response, and thus seroprevalence underestimates total population-based exposure to HPV. Information regarding HPV seroprevalence among MSM in Brazil is limited, and a greater knowledge of HPV seroprevalence is needed to develop recommendations for use of a HPV vaccine. Past studies of the Brazilian MSM population have used convenience sampling to recruit study participants [12], [13]. To minimize selection bias, this study utilized an alternative method, respondent-driven sampling (RDS), which is useful for recruiting subjects among hidden populations such as MSM. RDS [14], [15] is a type of chain-referral sampling or snowball sampling recruitment methodology, and “is based on the recognition that peers are better at locating and recruiting other members of a hidden population than outreach workers and researchers” [16]. RDS is based on a model that takes into account the network size of participants and recruitment patterns [17], [18], [19]. Here, RDS was used to estimate rates of infection and co-infections for HIV-1, syphilis, HBV, HCV, HPV-16/18, and HTLV among a group of MSM in Campinas, São Paulo, Brazil.

## Methods

### Ethics Statement

The protocol to study these prevalence in individuals from all age groups was approved by the Institutional Review Board of the Population Council in the U.S., the ethics committee of Campinas State University (Unicamp), and the National Ethics Committee, Conselho Nacional de Ética em Pesquisa (CONEP) in Brazil. All individuals enrolled in this study provided their signed informed consent. Moreover, individuals aged 14 to 17 years were asked also to provide parental consent; however, parental consent was not required if an adolescent felt that requesting parental permission would threaten his well-being. This consent procedure was approved by the ethics committees. Consent forms were kept separately from questionnaires, recruitment coupons, and biological samples so names could not be linked to any study data collected.

### Study population

This study was conducted in Campinas, Brazil, a city 100 km distant from São Paulo. Campinas metropolitan area (about one million people) is the 14^th^ most populous in Brazil and the 3^rd^ in the São Paulo State. Currently, the city concentrates about one third of the industrial production of the São Paulo state and is an administrative and cultural centre. Campinas is a referral pole for health care for 19 municipalities encompassing approximately 3,600 square kilometers and 2.33 million inhabitants. Reasons for undertaking the study in Campinas included that it had an important gay community and a well-organized AIDS municipal program that notifies AIDS cases occurring in the city. Here, the RDS method was used to recruit 658 MSM in the metropolitan area of Campinas between October 2005 and October 2006, as previously described [19]. Briefly, the recruitment seeds were selected by partnering with non-governmental organizations in the gay movement and during the Gay Pride Parade, based on their large social network. Each seed received three unique, non-replicable, recruitment coupons to give to peers who also fit the eligibility criteria for the study. Individuals were considered eligible if they were aged ≥14 years, resided in one of the 19 municipalities of the metropolitan area of Campinas, had oral or anal sex with a man within 6 months prior to the study, had arrived at the study site with a valid study recruitment coupon, and were not under the obvious influence of drugs and/or alcohol at the time of enrollment. Seed participants provided a blood sample to test for syphilis and HIV-1 seropositivity using a rapid test, and received up to three coupons to invite their peers to participate in the study. This process was repeated for one year. Study participants were also offered vaccination against hepatitis B virus. Of the 658 subjects who participated in this study, 585 subjects agreed to provide a second blood sample to test for additional infectious diseases.

### Serological tests

Serum samples obtained from 558 individuals previously examined for syphilis and HIV-1 by rapid tests [20] were included in this study. Briefly, syphilis was detected by the Abbott Determine Syphilis TP rapid POC test (Abbott Diagnostics, UK). Detection of anti-HIV-1 was performed using the Abbot Determine HIV-1/2 test and the HIV-1/HIV-2 Rapid Test (Bio-Rad Laboratories, Redmond, WA, USA). In case of discordance between the two test results, the Uni-Gold Recombigen HIV test (Trinity BioTech, Bray, Ireland) or the Rapid test HIV-1/2 (Bio-Manguinhos, FIOCRUZ) was used as a tiebreaker. To identify recent HIV-1 infections, anti-HIV-1 positive samples were further subjected to a quantitative competitive capture enzyme immunoassay (BED-CEIA) to determine the proportion of anti-HIV-1 specific IgG in relation to the total IgG, as previously described [21]. Detection of HBsAg, anti-HBc, and anti-HBs was performed using an enzyme-linked immunosorbent assay (ELISA) (Hepanostika Uni-form Organon Teknika B.V., Boxtel, Holland). Antibodies against HCV and HTLV-1/2 were detected using commercially available ELISA tests (Radim SpA Diagnostic, Italy and Symbiosys Diagnóstic, São Paulo, Brazil, respectively). Serum samples were also tested for anti-HPV types 16 and 18 virus-like particles using an ELISA specific for each HPV type, as previously described [22].

### Data Analysis

Data analysis was conducted using sample proportions and population-based estimates with 95% confidence intervals (CI) for all STIs described. Data was weighted for personal network size and recruitment patterns based on RDS Analysis Tool version 5.6 (RDSAT). Also, some sociodemographic and behavioral profile information for STI-positive cases was described, and these cases were plotted to examine network structures using a network illustration program (NetDraw 2.3.1).

## Results and Discussion

Methods used to access and analyse serological surveys in hidden populations such as MSM include snowball sampling, time location sampling, and RDS analysis. Few studies have been done comparing their relative advantages. A recent simulative comparison study with HIV infected individuals in Fortaleza, Brazil, with these methods, favors toward RDS because it achieved the sample size faster and at lower cost [23]. RDS is a relatively recent method (empirical studies started in 2003), now conducted in studies from all five continents [24]. However, some methodological questions about the sampling process itself, such as the precise estimation of the design effect and more adequate tools for data analysis produced by RDS, have not yet been tackled. Also, there is no consensus to the best estimator, especially for dispersion measurements (variance and confidence intervals), to be used and the best analytical approach to estimate association measurements that take into account data structure and homophile. Therefore, we chose a more conservative approach, presenting bi-varied analyses of the association between HIV infection and other STI infections. Even these should be cautiously considered given the lack of consensus about variance measurements by the present estimators in use.

**Figure 1:**
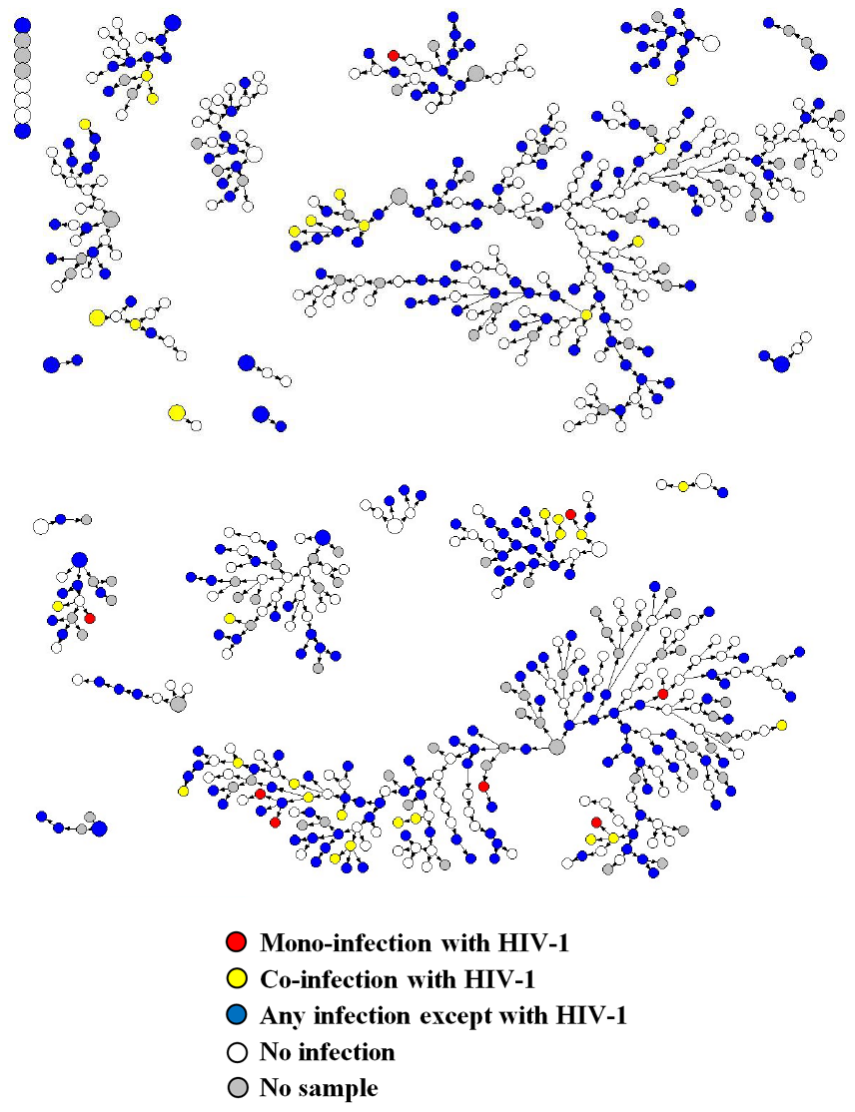
Recruitment network indicated by the presence of HIV-1 mono- infection (red circle), HIV-1 co-infections (yellow circles) and any other infection (blue color).

A sociodemographic profile of the studied population is shown in Table 1. There were no significant differences in demographic characteristics of the participants when analyzed by total sample percentages (crude values) or when the values were adjusted for personal network size and recruitment patterns (analysis in RDSAT). Figure 1 shows a representation of the recruitment network, indicating cases of mono-infection with HIV-1, and also co-infections with HIV-1 and any other infection. The study results showed that after RDSAT adjustment, 57.1% of individuals were aged <24 years (median age of 23 years), and 28.7% were aged <19 years. More than 50% of the MSM group had attended high school. Skin color was assessed by self-report (∼50% claimed white, and ∼33% claimed brown or mulatto), and the results reflected the miscegenation of the Brazilian population. Most individuals (∼70%) claimed an exclusively homosexual orientation and had more than one sexual partner during the preceding two months. Only 1% of the participants reported use of injecting drugs, and only 3% had received a blood transfusion. Recruitment using RDS methodology did not lead to significant differences in seroprevalence of study infections, regardless of whether the data was analyzed by total sample percentages (crude values) or in RDSAT (Table 2).

**Table 1:** Sociodemographic characteristics of the studied MSM group.

Variables	Crude^a^	Adjusted by RDSAT^b^	
	%	%	95% CI
**Age (years; n = 658)**			
14–19	25.4	28.7	22.5–35.0
20–24	28.9	28.4	23.4–33.7
25–34	20.4	19.7	15.4–24.7
35–63	25.4	22.5	16.8–28.1
Median age (IQR)	23 (19–30)	-	-
**Schooling degree (n = 657)**			
Some/completed primary/secondary	15	16.7	12.2–21.7
Some/completed high school	55	53.2	47.8–59.3
Some/completed college	30	30.1	24.1–35.9
**Skin color (n = 644)**			
White	56	54.4	48.5–59.8
Brown/mulatto	35	36.9	31.6–42.5
Black	6	5.3	2.9–8.3
Other	3	3.4	1.7–5.7
**Sexual orientation (n = 658)**			
Homosexual	73.1	67.7	62.0–73.4
Bisexual	23.3	26. 7	21.6–31.8
Other	3.6	5.6	2.3–9.6
**Ever received payment for sex (n = 636)**	16.8	14.7	10.8–19.5
**Sexual relations the last 2 months (n = 603)**			
Only MSM	86.4	82.4	75.8–88.2
MSM and women	13.6	17.6	11.8–24.2
**Number of sexual partners last 2 months (n = 601)**			
1 partner	30.3	33.8	27.5–39.5
>1 partner	69.7	66.2	60.5–72.5
**Unprotected receptive anal intercourse in the last 2 months (n = 631)**			
No	59.4	62.5	57.1–67.7
Yes	40.6	37.5	32.3–42.9
**Used any illicit drug past 6 months (n = 616)**	33.4	31.2	20.7–39.5
**Ever injected drugs (n = 630)**	2.0	1.0	0.4–2.9
**Ever blood transfusion (n = 631)**	4.1	3.0	1.7–4.5

Sample sizes vary slightly because of missing data.

a Sample percentages represent the proportion of the study population with their characteristics.

b Population-based estimates are based on analysis in RDSAT, which is adjusted for personal network sizes and recruitment patterns. IQR: Interquartile range.

**Table 2:** Prevalence of serological markers of infections not adjusted and adjusted by RDSAT.

Serological markers	Total (n = 558)	Crude	RDSAT	RDSAT
	n	%	%	CI 95%
**Anti-HPV-16**	188	33.7	31.9	26.1–38.1
**Anti-HPV-18**	138	24.7	20.3	15.1–25.6
**HBV** a	84	15.1	11.4	0.8–14.9
**Anti-HBc**	77	13.8	11.3	7.7–14.9
**HBsAg**	19	3.4	2.3	0.8–4.1
**Anti-HBc alone**	11	2.0	1.8	0.8–3.5
**Anti-HBs/Anti-HBc**	53	9.5	9.1	7.0–11.8
**Anti-HBs alone**	177	31.7	29.0	24.3–33.0
**Anti-TP**	62	11.1	10.0	6.6–13.6
**Anti-HIV-1**	41	7.4	7.6	4.3–10.9
**Anti-HTLV-1/2**	8	1.4	1.5	0.5–3.0
**Anti-HCV**	7	1.2	1.0	0.0–3.2

RDSAT analysis adjusted for network sizes and recruitment patterns,

a HBV markers of infection excluding anti-HBs alone.

As expected, the highest infection prevalences were found for HPV-16 and HPV-18 (31.9% and 20.3% after adjustment in RDSAT, respectively).

Due to a lack of commercial detection tests that can be used for serum samples, only a few serology studies of HPV have been conducted to date. To our knowledge, our study is the first to determine the HPV seroprevalence of a MSM population in Brazil. A previous multicenter study conducted using male serum samples without regard to sexual orientation and the same in-house test used in our study showed lower prevalences of both HPV-16 and 18 (11.2%, and 5.8%, respectively) [25]. Several studies have utilized PCR to examine the prevalence of anal or genital HPV infection in MSM. Unlike serology, which is a measure of past exposure to HPV, PCR only detects current infection. Another recent study conducted with MSM in Argentina and using RDS methodology for recruitment showed an overall anal HPV DNA prevalence of 83.5%, and a specific HPV-16 prevalence of 17.3% [26]. A study conducted in China showed an anal HPV-16 prevalence of ∼13% in a HIV-1 positive MSM group [27]. A recent study conducted in the same geographic region as our study showed a much lower prevalence of HPV-16/18 (13%) in an MSM group [28] than our study, where the overall prevalence of HPV-16/18 was 40.3%, and the specific prevalences of HPV-16 and 18 were 33.7% and 24.7%, respectively. These differences may be associated with the methods used to detect HPV infection (antibody detection in our study vs. DNA detection in most studies) or recruit subjects. Indeed, HPV seroprevalence reflects a history of HPV exposure. In a recent study [29], using an evaluation method similar to that employd in the present study, very high seroprevalences of HPV16 and HPV18 have been observed among non coinfected (30.8% and 21.7%, respectively) and HIV coinfected (56.2% and 38.0%) women living in Rio de Janeiro, Brazil, which is in agreement with the data obtained here.

In our study, >11% of the population was infected with HBV, and >2% were HBsAg positive at the time of blood sample collection (Table 2). These numbers are ∼10-fold higher than the overall prevalences of HBV infection and HBsAg positivity determined in the general population of a previous study conducted in the same geographical region [30]. Protective anti-HBs antibodies were found in 235/558 (42.1%; 40.1% after RDSAT adjustment) study subjects. However, only 177/558 (32%) displayed a serological profile compatible with a vaccine response (anti-HBs alone, Table 2). Moreover, three individuals displayed an unexpected profile, with simultaneous presence of HBsAg and anti-HBs (not shown). Guidelines published by the Brazilian Ministry of Health recommend that MSM should be immunized against HBV; however, we observed that the majority of MSM were not protected against HBV infection (individuals without any HBV marker of infection and without anti-HBs alone). Therefore, it is important for health care providers to educate and vaccinate their at-risk male patients against both hepatitis A and hepatitis B infection [31].

After adjustment in RDSAT, the infection rates for HIV-1 and syphilis in our study were 7.6%, and 10%, respectively (Table 2). These results are in agreement with a recent study conducted with a large MSM population (3,859 subjects) living in ten cities located in all five Brazilian geographic regions. The HIV-1 overall prevalence in MSM was estimated between 11.1% and 14.2%. HIV-1 prevalence ranged from 5.2% (Recife, Northeast Brazil) to 23.7% (Brasília, Central Brazil). Differences in HIV seroprevalence data into the Brazilian population may reflect differences in level of education, socioeconomic development and infrastructure in the different cities [32]. In the present study, 11 of 41 HIV-1 seropositive individuals (27.5%) were classified as recent seroconverters, corresponding to an estimated incidence rate of 4.88%/year (95% CI 2.00%–7.76%), which can be considered quite high. Indeed, in a previous study conducted by our group we found an estimated incidence of 1.68%/year (95% CI 1.26%–2.10%) for people seeking HIV diagnosis at Voluntary and Counselling Centers of Rio de Janeiro [33]. However, when these individuals were categorized by risk behavior, the estimated incidence among MSM was 11-fold higher than among heterosexual men (11.96% year (95% CI 6.10–17.82) vs. 1.12%/year (95% CI 0.55–1.68). This suggests that preventive actions aimed at the MSM community are insufficient, and a priority must be given to efforts focused on this group [33]. Among our study subjects, 7 men were infected with HCV and 8 were infected with HTLV. Contrary to the results of a recent study conducted in Brazil which showed a very high association between HTLV and hepatotropic viruses [34], in our study, HTLV infection was predominantly found as a mono-infection (5/8 individuals, 63%). In contrast, HCV only appeared as a co-infection with the other agents. These results indicate that the main routes of transmission for these two viruses (HCV and HTLV) may not have been shared in our study population. Indeed, it is known that HCV is mainly transmitted by blood, whereas HTLV is mainly transmitted mother-to-child through breast-feeding; followed by sexual transmission (predominantly from men to women), and transmission by cellular blood components [35].

Hepatitis C remains a major global health problem; however, since the initiation of blood screening requirements in most countries, transmission by blood or blood products in developed and many developing countries has been well controlled. The extent to which HCV is transmitted by sexual activity remains to be determined; however, accumulated epidemiologic evidence indicates that HCV can be transmitted by sex with an infected partner, most probably by mucosal exposure to infectious blood [6]. In general, there is a consensus that sexual activity is much less efficient than blood contact for transmitting HCV. It is known that the rate of HCV infection among seronegative drug users is currently increasing [36], and in San Francisco, the rate of new cases among drug users was recently reported to be increasing by ∼27% per year [37]. In the present study, six of the seven patients infected with HCV had used illicit drugs in the preceding 6 months (Table 3). This strong association between HCV infection and drug use makes us believe that in Brazil, as in other studied countries, injection drug users are mainly responsible for maintenance of HCV circulation. The clinical and social significance of HCV infection, along with the fact that HCV prevalence among homosexual drug addicts is increasing, is sufficient to warrant clinical trials to measure the efficacy of new HCV vaccines in the MSM population.

**Table 3:** Sociodemographic characteristics by infection agents.

Variables	HIV Total = 41 n (%)	Syphilis Total = 62 n (%)	HPV 16/18 Total = 224 n (%)	HBV Total = 84 n (%)	HCV Total = 7 n (%)	HTLV-1/2 Total = 8 n (%)
**Age (years)**						
14–19	4 (2.9)	7 (5.1)	32 (23.2)	10 (7.3)	0	2 (1.4)
20–24	10 (5.9)	9 (5.4)	66 (39.3)	13 (7.7)	2 (1.2)	1 (0.6)
25+	27 (10.7)	46 (18.3)	126 (50.0)	61 (24.2)	5 (1.9)	5 (2.0)
*p*-value	0.013	<0.0001	<0.0001	<0.0001	NS	NS
**Schooling degree**						
Some/completed prim/second.	13 (15.5)	16 (19.1)	33 (39.3)	22 (26.2)0	2 (2.4)	0 (0)
Some/completed high school	22 (7.2)	28 (9.2)	123 (40.2)	38 (12.4)	3 (0.9)	7 (2.3)
Some/completed college	6 (3.6)	18 (10.8)	68 (40.7)	23 (13.8)	2 (1.2)	1 (0.6)
* p*-value	0.003	0.047	NS	0.010	NS	NS
**Skin color**						
Black	4 (12.5)	9 (28.1)	14 (43.7)	7 (21.8)	1 (3.1)	1 (3.1)
Non-black	33 (6.4)	52 (10.1)	205 (39.9)	74 (14.4)	6 (1.2)	7 (1.4)
* p-value*	NS	0.005	NS	NS	NS	NS
**Sexual orientation**						
Homosexual	33 (8.2)	45 (11.1)	170 (41.9)	60 (14.8)	5 (1.2)	7 (1.7)
Bisexual	5 (3.8)	12 (9.1)	45 (34.1)	18 (13.6)	1 (0.7)	0 (0)
Other	3 (14.3)	5 (23.8)	9 (42.8)	6 (28.6)	1 (4.7)	1 (4.8)
* p*-value	NS	NS	NS	NS	NS	NS
**Ever received payment for sex**						
Yes	10 (11.4)	20 (22.7)	41 (46.6)	23 (26.1)	3 (3.4)	0 (0)
* No*	24 (5.4)	38 (8.5)	171 (38.3)	56 (12.6)	4 (0.9)	8 (0.2)
* p*-value	0.036	<0.0001	NS	0.002	NS	NS
**Sexual relations the last 2 months**						
Only MSM	30 (6.8)	49 (11.2)	190 (43.3)	67 (15.3)	5 (1.1)	6 (1.3)
MSM and women	4 (5.6)	7 (9.8)	16 (22.5)	9 (12.7)	1 (1.4)	2 (2.2)
* p*-value	NS	NS	0.001	NS	NS	NS
**Number of sexual partners last 2 months**						
1 partner	4 (2.6)	11 (7.2)	55 (36.2)	17 (11.2)	1 (0.6)	2 (1.3)
>1 partner	29 (8.2)	45 (12.6)	149 (41.8)	59 (16.6)	5 (1.4)	6 (1.7)
* p*-value	0.021	0.049	NS	NS	NS	NS
**Unprotected receptive anal intercourse in the last 2 months**						
No	20 (6.4)	32 (10.2)	114 (36.2)	40 (12.7)	5 (1.6)	5 (1.6)
Yes	16 (7.3)	26 (11.9)	99 (45.4)	37 (16.9)	2 (0.9)	3 (1.4)
p-value	NS	NS	0.02	NS	NS	NS
**Used any illicit drug past 6 months**						
** **Yes	12 (6.7)	25 (13.9)	71 (39.6)	28 (15.6)	6 (3.3)	6 (3.3)
No	20 (5.8)	33 (9.6)	139 (40.6)	44 (12.8)	1 (0.3)	2 (0.6)
* p-value*	NS	NS	NS	NS	0.008	0.015
**Blood transfusion lifetime**						
Yes	4 (20.0)	5 (25.0)	10 (50.0)	6 (30.0)	2 (10.0)	0 (0)
No	31 (6.1)	54 (10.5)	204 (39.6)	70 (13.6)	5 (0.9)	8 (1.5)
* p*-value	0.013	NS	NS	0.050	0.025	NS

All *p* values by Fisher's exact test (non-adjusted by sampling methodology).

More than 50% of our study population had at least one type of infection (Table 4), and co-infections were found in 29% of the studied population. HPV-16, HBV, and syphilis showed similar frequencies of mono-infection (55/188, 29%; 26/84, 31%; 25/62, 40%, respectively). HPV-18 and HIV-1 were more likely to be found in co-infections (mono-infection rates of 23/138, 17%, and 6/41, 15% respectively), while HCV was only found in co-infections. Additionally, both parenteral and STIs were generally more prevalent in HIV-1 positive individuals than in HIV-1 negative individuals (Table 5 and Figure 1). Oncogenic HPV-16 was the major co-infection agent identified among HIV-1 positive individuals, followed by infection with HPV-18 (Table 5). One previous report found that HPV-16 was the most prevalent HPV, and represented the most common type of HPV infection in the anal canal of both men and women, and also in HIV-infected and-uninfected MSM [38].

**Table 4:** Prevalence of mono-infections and co-infections.

Infection Agent	Total = 558 n (%)	Mono-infections n (%)	Co-infections n (%)
**HPV-16**	188 (33.7)	55/188 (29)	133/188 (71)
**HPV-18**	138 (24.7)	23/138 (17)	115/138 (83)
**HBV**	84 (15.1)	26/84 (30)	58/84 (70)
**Syphilis**	62 (11.1)	25/62 (40)	37/62 (60)
**HIV-1**	41 (7.4)	6/41 (15)	35/41 (85)
**HTLV-1/2**	8 (1.4)	5/8 (62.5)	3/8 (37.5)
**HCV**	7 (1.2)	0/7 (0)	7/7 (100)
**Any infection**	300 (53.7)	-	-
**Any co-infection**	160 (28.7)	-	-
**No infection**	258 (46.2)	-	-

Values non-adjusted by RDSAT.

**Table 5:** Co-infections between HIV positive and negative individuals.

Infection Agent	Total = 558 n (%)	HIV negative (n = 517)	HIV positive (n = 41)	*p*-value^a^
**HPV16**	188 (33.7)	163 (31.5)	25 (60.9)	<0.0001
**HPV18**	138 (24.7)	119 (23.0)	19 (46.3)	<0.001
**HBV**	84 (15.1)	68 (13.2)	16 (39.0)	<0.0001
**Syphilis**	62 (11.1)	54 (10.4)	8 (19.5)	0.07
**HTLV-1/2**	8 (1.4)	8 (1.5)	0	1.000
**HCV**	7 (1.2)	4 (0.8)	3 (7.9)	<0.009
**Any co- infection**	160 (28.7)	125 (24)	35 (85.4)	0.0001

a All *p*-values by Fisher's exact test (non-adjusted by sampling methodology).

HPV-16/18 and HBV seroprevalences were significantly associated with a subject age >25 years (Table 3). Educational level was inversely correlated with HIV-1, syphilis, and HBV prevalence, and Black Brazilian homosexual men had a higher prevalence of syphilis than other groups. Only HPV-16/18 infections were highly associated with unprotected receptive anal intercourse or exclusively homosexual relations which occurred 2 months prior to study enrollment. Finally, use of illicit drugs in the past 6 months was only associated with HCV and HTLV infections.

## Conclusions

All studied infections (HIV-1, HBV, HCV, HTLV-1/2, HPV-16/18, and *Treponema pallidum*) were detected in the MSM group, with prevalences ranging from 1.2% (HCV) to 34% (HPV-16). More than 50% of the study group had some type of infectious disease, and ∼30% had more than one type of infection. Co-infections were found with all infectious agents studied, and certain infections such as HBV and HIV-1 were ∼10-fold more prevalent in the MSM group than in the general population. Although blood banks now routinely test for blood transmitted diseases, it remains necessary to control infections such as HCV and HTLV in the environments of drug users.

It is noteworthy that in our study, only 32% of individuals had a serological profile compatible with having been vaccinated against HBV, even though vaccinations are recommended for this group, and a vaccine has been available for >30 years. It is also possible that a significant proportion of the MSM population has not responded to vaccination, and therefore remains susceptible to HBV infection. The high rate of new of HIV-1 infections indicates that preventive actions must continue to be a priority. Greater efforts should be made to educate individuals about their risk, and screening anoscopy may be advisable as part of routine clinical care for MSM. Additionally, HPV vaccination should be considered as a prophylactic measure to reduce the risk of anal cancer. Finally, genomic analysis of infectious agents should help to trace routes of dissemination within networks obtained by RDS methodology.
